# 2673. Clinical and Demographic Characteristics of Suspected and Confirmed Monkeypox Patients: An Observational Cohort Study

**DOI:** 10.1093/ofid/ofad500.2284

**Published:** 2023-11-27

**Authors:** Mariana A Kundro, Guillermo Viloria, Isabel Pastor, Marina Alonso Serena, Marcelo H Losso

**Affiliations:** Hospital JM Ramos Mejía, Buenos Aires, Ciudad Autonoma de Buenos Aires, Argentina; Hospital JM Ramos Mejía, Buenos Aires, Ciudad Autonoma de Buenos Aires, Argentina; Hospital JM Ramos Mejía, Buenos Aires, Ciudad Autonoma de Buenos Aires, Argentina; Hospital JM Ramos Mejía, Buenos Aires, Ciudad Autonoma de Buenos Aires, Argentina; Hospital JM Ramos Mejía, Buenos Aires, Ciudad Autonoma de Buenos Aires, Argentina

## Abstract

**Background:**

Monkeypox (mpox) infection is a re-emergent disease of public health concern worldwide.

**Methods:**

All the patients with suspected mpox infection attending a large public sexual health clinic in Buenos Aires, were included in this single center, observational cohort study. Confirmed case of mpox was defined as a positive real time PCR of either skin lesions or anal/oropharynx swab samples. We aimed to describe clinical features and evolution of confirmed cases and to compare characteristics of confirmed and unconfirmed individuals during a mpox outbreak in Argentina.

**Results:**

Of the 85 patients (96.47% male, 90.12 % MSM, mean age 34 years) with suspected mpox between July 2022 and March 2023, 60 (70.48%) had a confirmed diagnosis. All the patients presented skin and/or mucous rash with a median time from the onset of symptoms of 6.0 (IQR 4-7) days. Lesions were most commonly distributed on genital area (57% of confirmed cases), followed by perianal region in 37% of patients. Dysphagia was reported in 31.7% vs. 20% of confirmed and unconfirmed subjects respectively (p: 0.27) , whereas proctitis symptoms were more often in mpox cases (35.0 vs 12.0%; p: 0.01). Syphilis was diagnosed in 60% of the cohort. Compared to unconfirmed patients, mpox patients were more likely to be MSM (98.3% vs. 88%; p: 0.04) and PrEP users (50.0 vs. 18.7%; p:0.03) . We found no differences among groups regarding HIV status (30% vs. 36%; p: 0.58). Main alternative diagnoses in unconfirmed patients were syphilis in 44% and HIV infection in 8% of individuals.

Hospitalization rate was 4.25 % (5/85). Admissions in mpox group were due to: cellulitis (n:1), pain control (n:1), tonsillar abscess (n:1) and infectious mononucleosis/acute HIV coinfection (n:1). Other complications reported were penile edema (n:3) , skin sobreinfection (n:2), severe dysphagia (n:1) and conjunctivitis (n:1) One patient was admitted in the unconfirmed group due to severe sepsis. There were no fatal cases.

**Conclusion:**

We found that almost a third of participants had no confirmed diagnosis of mpox infection despite high clinical suspicion in this cohort. MSM and PrEP users were more likely to be diagnosed with mpox. The elevated rate of concomitant syphilis reflects that patients should always be tested for other STIs.

**Disclosures:**

**All Authors**: No reported disclosures

